# Current and Future Priorities for School Food and Nutrition Education in South Korea: Perspectives of Nutrition Teachers

**DOI:** 10.3390/nu18172829

**Published:** 2026-08-28

**Authors:** Seung Jae Lee, Jieun Oh, Kyung Won Lee

**Affiliations:** 1 Department of Food and Nutrition, Yongin University, Yongin 17092, Republic of Korea; sjlee@yongin.ac.kr; 2 College of Science & Industry Convergence, Ewha Womans University, Seoul 03760, Republic of Korea; oje96@ewha.ac.kr; 3 Department of Home Economics Education, Korea National University of Education, Cheongju 28173, Republic of Korea

**Keywords:** food literacy, school nutrition education, nutrition teachers, school meals, sustainable diets, nutrition curriculum, dietary behavior, qualitative research, South Korea

## Abstract

**Background/Objectives**: School-based food and nutrition education is an important public health strategy for developing healthy dietary behaviors during childhood and adolescence. However, limited qualitative evidence describes how nutrition teachers, who are simultaneously responsible for school meals, perceive established educational priorities and emerging needs. This study explored current and future priorities for school-based food and nutrition education in South Korea. **Methods**: Two online focus group interviews were conducted with eight nutrition teachers working in elementary, middle, and high schools. The interviews were transcribed and analyzed using qualitative content analysis procedures. **Results**: Current priorities included foundational nutrition and food-related knowledge, balanced eating, environmental issues, low-sodium and low-sugar diets, understanding school meals, prevention of eating disorders and chronic diseases, and table manners and picky eating. Future needs centered on food choice and food literacy, sustainable diets, interdisciplinary education, and understanding diverse food cultures. Across themes, participants described a shift from primarily knowledge-focused instruction toward applied dietary competence, a broadening from individual disease prevention toward health and sustainability, and stronger integration of classroom education with school meals and other subjects. **Conclusions**: From the perspectives of the participating nutrition teachers, the findings provide formative evidence for curricula and interventions that combine foundational nutrition science with food-choice skills, age-appropriate preventive messages, sustainable dietary practices, and opportunities for behavioral application in the school meal setting. Future quantitative and intervention studies, including nationally representative samples, are needed before robust national priorities can be established.

## 1. Introduction

Dietary behaviors established during childhood and adolescence are associated with growth, development, and health across the life course [1,2]. At the same time, young people make food decisions in environments characterized by the widespread availability of processed and ultra-processed foods, persuasive marketing, digital nutrition information, and persistent diet-related health concerns [3,4]. Supporting the development of healthy dietary behaviors during these life stages is therefore a public health priority, and schools provide repeated access to students during a period when knowledge, preferences, and eating practices are still developing [5,6,7,8].

School-based food and nutrition education can improve food consumption, dietary behaviors, and healthy eating practices among children and adolescents [5,6,7,8]. Its public health value, however, ultimately depends on whether students can effectively translate what they learn in the classroom into everyday food decisions. Knowing the functions of nutrients or the components of a balanced meal does not by itself ensure that students can interpret labels, compare foods, respond to marketing claims, or apply dietary guidance in actual eating situations. Qualitative evidence from teachers similarly indicates that food and nutrition education becomes more meaningful when instruction is explicitly connected to students’ daily lives and supported by practical learning opportunities [9].

Food literacy offers a useful framework for addressing this knowledge-to-action gap. It encompasses the knowledge, skills, and behaviors required to plan, manage, select, prepare, and eat food in ways that meet individual and social needs [10]. For school-aged children and adolescents, this includes not only foundational nutrition knowledge but also the ability to evaluate food information, make reasoned choices, and adapt decisions to health needs and real-life constraints [10,11]. These competencies are increasingly important in rapidly changing and highly commercialized food environments [11].

The scope of school nutrition education is also widening beyond individual disease prevention. Environmental sustainability is increasingly recognized as relevant to dietary guidance and nutrition education [12]. Students may therefore need to consider food waste, carbon impacts, animal welfare, local and seasonal foods, and the cultural meanings of food alongside nutritional adequacy. Because these issues involve health, environmental, economic, and social considerations, they favor interdisciplinary and experiential approaches rather than isolated, content-heavy instruction [11,13]. Recent competency-based approaches further emphasize interconnected food-systems competencies that enable young people to understand food choices within broader health, environmental, social, and economic contexts [13].

In South Korea, nutrition teachers occupy a distinctive position at the intersection of nutrition education and school foodservice, with a formally integrated professional role encompassing both school meal management and student dietary guidance. Under the Enforcement Decree of the School Meals Act, their duties include menu planning, food ingredient selection and inspection, hygiene and safety management, dietary guidance, information provision, nutrition counseling, and supervision of foodservice personnel [14]. Thus, unlike systems in which educational and foodservice functions are assigned to separate professionals, Korean nutrition teachers are institutionally positioned to connect students’ food and nutrition education with the operation of school meals. More broadly, evaluation of school-based food and nutrition education should extend beyond knowledge acquisition to include the development and application of food-related skills and behaviors [8]. Their responsibilities can connect classroom content with menus, meal service, students’ eating behaviors, and the daily operation of school meals. Previous Korean studies have examined dietary education and nutrition counseling in schools [15], and a recent focus group study explored the need for school-based dietary education from the perspective of home economics teachers [16]. However, qualitative evidence focused specifically on nutrition teachers across elementary, middle, and high schools remains limited, particularly regarding how they distinguish current educational priorities from future needs. This study therefore asked: (1) Which topics do nutrition teachers currently perceive as important in school-based food and nutrition education? (2) Which topics do they expect to become increasingly important? The study aimed to provide formative evidence for school nutrition curricula, teaching resources, professional development, and future interventions.

## 2. Materials and Methods

### 2.1. Study Design

This qualitative descriptive study used focus group interviews (FGIs) to explore nutrition teachers’ shared and contrasting perceptions of current priorities and future needs in school-based food and nutrition education. FGIs were selected because interaction among participants can elicit common experiences, clarify differences in practice, and prompt reflection on issues that may not emerge in individual interviews [17]. The data were analyzed using qualitative content analysis [18]. Manuscript reporting was guided by the Consolidated Criteria for Reporting Qualitative Research (COREQ) [19].

### 2.2. Participants and Recruitment

Participants were recruited among nutrition teachers across South Korea using purposive sampling. Eligible participants were currently employed nutrition teachers who had direct experience providing food and nutrition education in elementary, middle, or high schools, understood the study purpose and procedures, could communicate in Korean, and voluntarily consented to participation and audio recording. Individuals who did not consent to the interview or recording were excluded.

Eight nutrition teachers participated and were assigned to two focus groups of four participants each to facilitate focused group interaction while allowing sufficient opportunity for individual contributions. The participants included two teachers in their 20s, three in their 30s, and three in their 40s; one participant was a man and seven were women. Four worked in Gyeonggi Province, two in the Jeolla region, and two in Daejeon–Sejong. Teaching service ranged from 4 years or less to more than 8 years. Four participants worked in elementary schools, three in middle schools, and one in a high school. The sample included teachers from different school levels and regions to capture variation in educational experience; it was not intended to be statistically representative of all nutrition teachers in South Korea. Participant characteristics are presented in Table 1.

### 2.3. Focus Group Procedures and Data Collection

Before each FGI, participants received an open-ended pre-interview questionnaire concerning school-based food and nutrition education and were invited to organize their experiences and views in advance. The questionnaire was used to support reflection before the group discussion and was not analyzed as a separate data source. The two FGIs were conducted in Korean through Zoom (Zoom Video Communications, Inc., San Jose, CA, USA) on 29 August and 2 September 2024. Each session lasted approximately 90 min. At the beginning of each session, the moderator explained the study purpose, discussion procedures, voluntary nature of participation, confidentiality protections, and audio-recording process. All participants provided consent before recording began.

One researcher with experience in food and nutrition education research moderated both sessions using a semi-structured guide. The moderator introduced broad questions, used follow-up probes to clarify meaning and elicit concrete examples, encouraged quieter participants to contribute, and invited participants to respond to or contrast one another’s experiences. Field notes were recorded during or immediately after the sessions to document contextual observations and emerging analytic insights. The audio recordings were transcribed verbatim in Korean and de-identified by replacing personal information with participant codes.

The semi-structured interview guide used during data collection was developed with reference to previous Korean studies of school dietary education and nutrition counseling [15,20] and was reviewed by the research team for alignment with the study objectives. The guide covered participant characteristics; topics considered important in current school-based food and nutrition education; reasons these topics were emphasized; examples of educational practice; and topics expected to become more important in the future. It was used flexibly so that the moderator could pursue issues raised by participants while ensuring that the same core domains were addressed in both groups. The focus group interview guide is provided in Appendix A.

### 2.4. Data Analysis

The Korean-language transcripts and field notes were analyzed using qualitative content analysis [18]. The research team followed an iterative six-step process. First, the transcripts were read repeatedly to understand the overall context and record initial observations. Second, passages relevant to the research questions were identified as meaning units. Third, the meaning units were condensed while preserving their contextual meaning and assigned initial codes. Fourth, codes with similar or related meanings were compared within and across the two groups and clustered into preliminary categories. Fifth, the categories were integrated into higher-order themes representing current educational priorities and future educational needs. Sixth, the thematic structure was reviewed against the coded excerpts, complete transcripts, and research questions, after which representative quotations were selected. The researchers cross-checked the emerging codes, categories, and themes, and differences in interpretation were resolved through discussion. Common patterns, differences between the two groups, and less frequently expressed or divergent views were retained when they contributed to interpretation. During analysis, the researchers also considered the extent to which themes were represented across participants and the two focus groups. Themes discussed across participants or groups were interpreted as more broadly shared perspectives, whereas themes raised by only a small number of participants were retained when analytically relevant but interpreted as emerging perspectives. Quotations were translated into English only after the Korean-language analysis had been completed, with priority given to preserving the original meaning and tone rather than literal word-for-word equivalence. Because the study followed a pre-specified two-group design, formal saturation criteria were not applied to determine sample size. This approach was chosen because the study was designed as a qualitative descriptive study using content analysis, with two focus groups specified a priori to obtain focused accounts from nutrition teachers and to compare shared and contrasting perspectives across groups.

### 2.5. Trustworthiness and Ethical Considerations

Several procedures were used to strengthen the credibility and dependability of the findings. The research team cross-checked the analysis by comparing meaning units, codes, categories, and themes with the original transcripts and discussing differing interpretations until agreement was reached. The research team had academic and research experience in food and nutrition education. Throughout the analytic process, the researchers remained attentive to how their disciplinary backgrounds and prior experiences might shape interpretation, and this reflexivity was incorporated into discussions of differing interpretations and reviews of the findings against the original transcripts. Field notes were considered during interpretation to retain contextual information from the group discussions. After the preliminary findings had been organized, participants were invited to review the interpretation; clarifications or corrections were incorporated where necessary. These procedures were intended to reduce reliance on a single researcher’s interpretation and to ensure that the final themes remained grounded in participants’ accounts.

Ethical safeguards were implemented throughout the study. The study was approved by the Institutional Review Board of Yongin University (IRB No. 2-1040966-AB-N-01-2407-HR-352-2). Before the interviews, participants received written and verbal information about the study purpose, procedures, expected duration, audio recording, confidentiality, voluntary participation, and the right to withdraw at any time without disadvantage. Written or electronic informed consent was obtained from all participants. Identifying information was removed from the transcripts, participant codes were used in all analytic files and quotations, and the data were stored in access-restricted files in accordance with the approved protocol.

## 3. Results

### 3.1. Current Educational Priorities in School-Based Food and Nutrition Education

Six themes described the topics that participants currently considered important for supporting students’ dietary behaviors and health: (1) basic nutrition and food knowledge and balanced eating; (2) environment and dietary practices; (3) low-sodium and low-sugar diets; (4) understanding school meals; (5) prevention of eating disorders and chronic diseases; and (6) table manners and reducing picky eating (Table 2). These themes were treated as qualitative categories rather than ranked by frequency. Across themes, participants repeatedly emphasized that educational content should support students’ ability to apply it in everyday eating situations.

Foundational nutrition and food knowledge, together with balanced eating, constituted a central area of current education. Participants identified nutrient types and functions, food groups, and the Korean Food Balance Wheel as core educational content. They viewed this knowledge as a basis for balanced eating and healthier food choices, but emphasized that instruction should lead to changes in attitudes and behaviors rather than end with factual learning.

*“I think the most important aspect of food and nutrition education is that students’ attitudes and behaviors change through basic knowledge, such as nutrients and the Food Balance Wheel.”*

(Participant L)

*“The most important topic is helping students develop balanced eating habits based on an understanding of nutrients.”*

(Participant J)

*“I focused on teaching basic content, such as how adolescents should compose meals and why they should eat a variety of foods, and on helping them put this into practice.”*

(Participant K)

*“I considered it important that the content could be applied in daily life rather than remaining theoretical.”*

(Participant I)

The relationship between environmental sustainability and dietary practices was another topic currently emphasized. Participants described education linking food choices with environmental sustainability, including carbon footprints, vegan diets, food miles, food waste, animal welfare, and low-carbon eating. Several teachers used activity-based lessons or planned to integrate environmental and nutrition content.

*“I think education on sustainable diets and environmental issues is important.”*

(Participant J)

*“I frequently taught lessons on carbon footprints, vegan diets, food miles, and food waste.”*

(Participant O)

*“I am planning education that links the environment and nutrition, including animal welfare and low-carbon practices.”*

(Participant P)

*“I conducted activity-based lessons on foods that reduce carbon emissions, focusing on the environment, health, and consideration for others.”*

(Participant M)

Low-sodium and low-sugar diets were viewed as important preventive topics. Participants emphasized lessons that would help students translate awareness of excessive sodium and sugar intake into daily dietary practices.

*“I considered lessons on low-sugar and low-sodium diets, as well as food waste, to be important.”*

(Participant O)

*“I provide education on low-sodium meals and reduced sugar intake for students in the middle elementary grades.”*

(Participant P)

Understanding school meals was identified as particularly relevant for elementary school students encountering institutional foodservice for the first time. Education focused on the concept, purpose, and operation of school meals.

*“Because elementary school students encounter institutional foodservice for the first time, I teach mainly about the concept of school meals and how they work.”*

(Participant O)

The prevention of eating disorders and chronic diseases was also considered an important component of current education. Participants perceived increasing needs for education on obesity, eating disorders, and chronic disease prevention, with the content adapted to students’ developmental stage. For older students, they also considered how to explain dietary management for specific health conditions in accessible terms.

*“Obesity and eating disorders have become serious issues among middle school students, so I think these topics need to be emphasized.”*

(Participant K)

*“I think education on chronic disease prevention is needed in relation to problems associated with sugar and sodium intake.”*

(Participant N)

*“For high school students, I considered how to make dietary management for different diseases easy to understand.”*

(Participant I)

Table manners and reducing picky eating were considered especially important for younger elementary school students. Participants linked picky eating not only to dietary imbalance but also to food waste in the school meal setting.

*“For younger elementary school students, I focus on table manners and preventing picky eating.”*

(Participant P)

*“Education is important because picky eating leads to food being discarded, and table manners are also necessary.”*

(Participant N)

Taken together, the current themes reflected three related public health nutrition functions. Foundational knowledge supported balanced eating and informed food choices; preventive topics addressed age-specific dietary and health concerns; and school meal-related topics provided recurring opportunities to practice food variety, table manners, and waste reduction. The consistent emphasis on application indicated that participants did not view knowledge and behavior as separate educational goals.

### 3.2. Future Educational Needs in School-Based Food and Nutrition Education

Four themes represented future educational needs: (1) food choice and food literacy; (2) sustainable diets and environmental sustainability; (3) interdisciplinary education; and (4) understanding diverse food cultures (Table 3). Food choice and environmental sustainability generated the most elaborated discussion, whereas interdisciplinary and cultural topics were raised less extensively and should be interpreted as emerging directions rather than broadly shared priorities.

Food choice and food literacy emerged as a major area of future educational need. Participants expected education that develops students’ capacity to make informed food choices to become increasingly important. Specific needs included interpreting food and nutrition labels, understanding processed and ultra-processed foods and food additives, recognizing local and seasonal foods, and using practical methods to estimate nutrient content.

*“In the future, it will be important to teach students how to make appropriate food choices.”*

(Participant I)

*“I think food literacy education is important because basic knowledge plays an important role when choosing foods.”*

(Participant L)

*“As ultra-processed foods have become more common, I think more education is needed to understand food additives.”*

(Participant O)

*“I also thought students need easy ways to calculate nutrient amounts.”*

(Participant I)

*“As environmental changes alter the availability of local and seasonal foods, I think these issues should also receive greater attention.”*

(Participant J)

Sustainable diets and environmental sustainability were also consistently identified as areas that would become increasingly important. Sustainable food education was viewed as both an existing topic and a field requiring further expansion. Participants emphasized low-carbon diets, carbon-footprint reduction, food waste, animal welfare, responsible consumption, and explicit links between food and environmental education.

*“Education on sustainable diets linked to the environment needs to be further emphasized.”*

(Participant J)

*“Through food education linked to environmental education, students need to recognize the connection between food and the environment.”*

(Participant P)

*“Low-carbon diets and environmental education will be important topics in the future.”*

(Participant K)

*“Food waste and animal rights also need to receive greater attention.”*

(Participant O)

In addition, participants highlighted the potential value of interdisciplinary approaches that connect food and nutrition education with other school subjects. Such integration was considered important for helping students understand food-related issues in broader scientific, social, and practical contexts.

*“I think we need integrated nutrition education connected with other school subjects.”*

(Participant J)

Finally, the increasing cultural diversity of Korean schools was perceived to require greater attention to diverse food cultures and ingredients. Participants considered this education important for fostering cultural understanding.

*“As multiculturalism increases, education should address diverse food cultures and ingredients.”*

(Participant J)

Comparison of the current and future themes revealed both continuity and expansion in the public health role of school nutrition education. Environmental sustainability appeared in both sets, suggesting further development of an existing area rather than the introduction of an entirely new topic. Food literacy extended the established emphasis on nutrition knowledge toward the use of information in real food-choice situations. Interdisciplinary and culturally responsive education further broadened the expected scope of school nutrition education, although these directions were less extensively represented in the discussions.

## 4. Discussion

This qualitative study provides formative public health nutrition evidence on how nutrition teachers perceive the current priorities and future needs of school-based food and nutrition education. Rather than representing a simple list of topics, the findings indicate three related shifts: from factual nutrition instruction toward applied dietary competence; from disease prevention alone toward the health, environmental, and social dimensions of eating; and from stand-alone lessons toward an integrated school nutrition environment that connects classroom learning with school meals and other subjects.

The first shift was evident in participants’ continued emphasis on nutrients, food groups, and balanced meals alongside their insistence that students should be able to use this knowledge in daily life. Nutrition knowledge remains an essential foundation, but it is not equivalent to the capacity to make and sustain health-promoting dietary choices. In this context, nutrition knowledge refers to factual understanding of nutrients and dietary principles, whereas nutrition literacy involves the ability to access, understand, and use nutrition information. Food literacy is broader, encompassing the knowledge, skills, and behaviors involved in selecting, preparing, and consuming food. Practical dietary competence refers here to the ability to apply these forms of knowledge and literacy to everyday food choices and eating situations [10,11]. Evidence from school interventions supports experiential, behavior-oriented, and contextually relevant approaches [8,21], while food literacy integrates knowledge with the skills and practices required to manage, select, prepare, and consume food [10]. The present findings therefore suggest that foundational nutrition content should be retained but intentionally framed as a resource for action rather than presented as an isolated body of facts.

The current emphasis on reduced sodium and sugar intake, prevention of obesity, eating disorders and chronic diseases, and reduction in picky eating demonstrates the preventive role that participants attributed to school nutrition education. Evidence linking excessive free-sugar intake with weight gain and high sodium intake with elevated blood pressure supports the public health relevance of these topics [22,23]. At the same time, content and communication should be adapted to developmental stage. Messages about body weight, dietary restriction, and disease risk require particular care during adolescence and should promote balanced patterns, body respect, critical interpretation of health information, and appropriate help-seeking rather than stigma or fear. For younger children, repeated exposure to varied foods and supportive meal routines may be more appropriate than emphasizing pressure-based messages about picky eating.

The future emphasis on labels, processed and ultra-processed foods, food additives, seasonal foods, and nutrient estimation illustrates the competencies required in contemporary food environments. Food literacy interventions may strengthen nutrition knowledge and self-regulatory capacities among adolescents [24]. Consistent with participants’ emphasis on food information and practical food-choice skills, food literacy education could provide opportunities for students to interpret food labels, compare food options, and apply nutrition information to everyday food choices. Taken together, these topics can be viewed as complementary components of healthy dietary behavior: sodium and sugar reduction and obesity prevention address preventive nutrition, food labels and ultra-processed foods support informed food choice and food literacy, and sustainable diets extend dietary decision-making to environmental and social considerations. Integrating these components may help students connect nutrition knowledge with practical dietary decisions in everyday contexts.

A second broadening concerned the environmental and social dimensions of dietary behavior. Participants identified sustainability among both current and future priorities, indicating their perception that it is already being introduced and may become more prominent in school-based food and nutrition education. Participants referred to carbon footprints, low-carbon diets, food miles, food waste, vegan diets, and animal welfare, consistent with recommendations that dietary guidance consider environmental sustainability alongside health [12,25]. School nutrition education should nevertheless avoid reducing sustainability to a single indicator. Nutritional adequacy, environmental impact, cost, access, feasibility, and cultural acceptability may not always align, and students need support in recognizing these trade-offs [13,25]. Food waste reduction may provide an especially concrete entry point because it links public and environmental health with behaviors observable in the school setting.

School meals emerged as an important setting for translating educational content into repeated dietary practice. Participants’ accounts positioned meals as more than a foodservice program: students encounter varied foods, practice table manners, respond to unfamiliar dishes, and observe the consequences of waste during daily meal service. School food-environment policies and meal strategies can influence dietary behavior and consumption [6,26,27]. Deliberate alignment between classroom instruction and the school meal setting could therefore provide opportunities for students to apply nutrition knowledge and food-choice skills in everyday eating situations. Nutrition teachers are particularly well positioned to connect these domains because their responsibilities span nutrition expertise, meal operations, and education [14]. This dual professional role may have influenced participants’ emphasis on food waste, table manners, school meals, and practical application, as these issues are directly encountered in both educational and meal-service settings. These findings should therefore be interpreted in the context of nutrition teachers’ combined responsibilities for education and school meal management.

Interdisciplinary and culturally responsive education was discussed less extensively but broadens the potential public health relevance of the findings. Food-related decisions involve science and numeracy, environmental and social systems, identity, and cultural inclusion. A curriculum-wide approach may allow students to revisit these ideas across multiple contexts [28]. Similarly, the increasing cultural diversity of Korean schools supports education that presents diverse ingredients and food practices respectfully. Because these themes were raised by relatively few participants, however, further stakeholder research is needed before they are treated as established national priorities.

The findings have practical implications for the design of curricula and school-based nutrition interventions. Learning objectives should be expressed as observable capabilities, such as interpreting a nutrition label, comparing foods for a stated health purpose, planning a balanced meal, or proposing feasible strategies to reduce food waste. Activities should use realistic food situations and be differentiated by school level. For example, students could compare the nutrition labels of commonly consumed foods, select an appropriate option based on specified nutritional criteria, and explain the reasoning behind their choice. Such activities can directly link nutrition knowledge with food-choice skills in everyday situations. In this way, nutrition education can address both individual dietary behavior and the broader contexts in which food choices are made.

Implementation will require support beyond individual lesson plans. Nutrition teachers need evidence-based and regularly updated materials, particularly for rapidly changing topics such as ultra-processed foods, food additives, and sustainable diets. Collaboration time with home economics, science, social studies, and environmental education teachers may make interdisciplinary work more feasible. Evaluation could distinguish among knowledge, self-reported skills, and observed behavior. Knowledge could be assessed through nutrition-related questions, self-reported skills through students’ perceived confidence in interpreting nutrition information and making food choices, and observed behavior through food-choice tasks or practices observed in the school meal setting. These outcomes would more directly assess the applied competencies identified by participants.

A key contribution of this study is its focus on professionals who work simultaneously with nutrition education and the operational reality of school meals. The inclusion of elementary, middle, and high school settings allowed the discussions to reflect developmental variation, while the focus group format enabled participants to build on or contrast one another’s experiences. Comparing current priorities with future needs also distinguished areas of continuity, such as sustainability, from areas of expansion, such as applied food literacy. The findings should therefore be understood as formative evidence for curriculum and intervention development rather than as an estimate of national prevalence.

Several limitations constrain interpretation. The study included only eight teachers in two focus groups from three broad geographic areas, and the school-level distribution was uneven, with only one high school teacher. Participation may have favored teachers with stronger interest or experience in food education. Group interaction can stimulate reflection but can also encourage conformity or reduce disclosure of dissenting views. The online format may also have influenced interaction dynamics and limited the observation of some nonverbal cues compared with in-person focus groups, although it facilitated participation across geographically dispersed locations. The pre-interview questionnaire may have helped participants prepare but may also have primed particular topics. The study relied on self-reported perceptions rather than observation of teaching or meal practices and included only nutrition teachers; therefore, the perspectives of students, families, school administrators, and professionals from other disciplines were not captured. This may limit a broader understanding of school food and nutrition education across the school community. In addition, the South Korean cultural, educational, and organizational context may limit the transferability of the findings to other educational systems. The study explored participants’ perceptions and priorities but did not evaluate the implementation or effectiveness of the educational strategies discussed. Translating selected quotations into English may also have reduced linguistic nuance despite efforts to preserve meaning.

Future studies should examine whether these priorities are shared across a larger and more diverse national sample and should include rural and urban settings, different school levels, and varied employment contexts. Additional qualitative work could investigate barriers to implementation, tensions between educational and foodservice responsibilities, and negative or divergent cases. A national survey could then estimate the prevalence and relative priority of the themes, while intervention studies could test whether curricula integrating food literacy, preventive nutrition, sustainable dietary practices, and school meals improve students’ dietary reasoning and behavior. Multiple stakeholder perspectives will be essential for developing and evaluating a coherent school nutrition education framework. Longer-term studies could further examine whether these changes are sustained and develop validated measures of food literacy, food decision-making, critical thinking, and sustainability-related competencies. Emerging digital environments, including social media and AI-based educational tools, also warrant investigation regarding their educational potential and influence on students’ food information and choices. Comparative research across curricular and policy contexts, together with studies of continuing education for nutrition teachers, could further inform future school food and nutrition education.

## 5. Conclusions

Nutrition teachers perceived school-based food and nutrition education as a public health function that should move beyond factual instruction toward applied dietary competence. Current priorities combined foundational nutrition knowledge with age-appropriate prevention, environmental issues, and learning connected to school meals, while future needs emphasized food literacy, sustainable diets, interdisciplinary learning, and diverse food cultures. From the perspectives of the participating nutrition teachers, the findings suggest that school nutrition curricula could integrate nutrition science with practical food-choice skills and use school meals as a setting in which students can apply healthy and sustainable dietary practices. This study offers formative evidence for curriculum and intervention development, but its small qualitative sample does not establish national prevalence or priority. Larger surveys, implementation research, and intervention studies are needed to determine feasibility and effects on students’ dietary reasoning and behavior. Nevertheless, these findings may provide preliminary evidence to inform future discussions on school food and nutrition education curricula and related policies in South Korea.

## Figures and Tables

**Table 1:** Characteristics of focus group participants.

Variables		Total (*n* = 8)
Age	20s	2 ^1^
	30s	3
	40s	3
Gender	Men	1
	Women	7
Region	Gyeonggi	4
	Jeolla	2
	Daejeon-Sejong	2
Number of years of continuous service	≤4 years	1
	4 < years ≤ 6	2
	6 < years ≤ 8	4
	>8 years	1
Working school	Elementary school	4
	Middle school	3
	High school	1

^1^ *n*, number of participants.

**Table 2:** Summary of focus group interview results on current topics in school dietary and nutrition education.

Contents	Key Points by Section
Basic nutrition and food knowledge and balanced dietary practices	Basic information about nutrients and food groups
	Food Balance Wheel and balanced diets
	Developing healthy dietary habits
	Practical application of nutrition knowledge to daily life
Environment and dietary habits	Sustainable dietary practices
	Carbon footprint and low-carbon diets
	Food mileage
	Food waste reduction
	Vegan diets and animal welfare
Low-sodium and low-sugar diets	Reducing sodium intake
	Reducing sugar intake
Understanding school meals	Understanding the concept of school meals
	Understanding the school meal system and operation
Prevention of eating disorders and chronic diseases	Prevention of obesity and eating disorders
	Healthy eating for chronic disease prevention
	Dietary management according to health conditions
Table manners and reducing picky eating	Developing proper table manners
	Preventing picky eating
	Promoting healthy eating behaviors among younger students

**Table 3:** Summary of focus group interview results on emerging topics in school dietary and nutrition education.

Contents	Key Points by Section
Food choice (e.g., food labels, nutrition facts labels, processed foods, food additives, seasonal foods)	Interpretation and use of food labels and nutrition facts labels
	Food literacy
	Understanding processed and ultra-processed foods (UPFs)
	Understanding food additives
	Understanding seasonal foods
Sustainable diets and environmental sustainability	Sustainable dietary practices
	Low-carbon diets and carbon footprint reduction
	Food waste reduction
	Animal welfare and responsible food choices
Interdisciplinary education	Integration of dietary and nutrition education across school subjects
	Interdisciplinary approaches to dietary and nutrition education
Understanding food cultures in multicultural families	Understanding diverse food cultures and food ingredients
	Promoting cultural awareness and inclusiveness

## Data Availability

The qualitative data generated in this study are not publicly available due to participant confidentiality and ethical considerations. Further inquiries may be directed to the corresponding author.

## Abbreviations

The following abbreviation is used in this manuscript:

FGI	Focus group interviews

## Appendix A

**Table A1:** Focus Group Interview Guide.

Theme	Interview Questions and Prompts
1. Current priorities	- What topics are currently considered most important in school-based food and nutrition education? - Please identify three to five topics.
2. Future priorities	- What additional topics would be helpful to address in future school-based food and nutrition education, or which topics are expected to become increasingly important? - Please identify three to five topics.

Note: During the focus group discussions, follow-up probes were used as needed to clarify participants’ responses and encourage elaboration.
